# Acute Kidney Injury in COVID-19: 90 Days of the Pandemic in a Brazilian Public Hospital

**DOI:** 10.3389/fmed.2021.622577

**Published:** 2021-02-09

**Authors:** Welder Zamoner, Camilla Andrade da Silva Santos, Luís Eduardo Magalhães, Paula Gabriela Sousa de Oliveira, André Luis Balbi, Daniela Ponce

**Affiliations:** Botucatu School of Medicine, University São Paulo State-UNESP, Botucatu, Brazil

**Keywords:** acute kidney injury, COVID-19, dialysis, risk factors, mortality

## Abstract

Renal involvement is frequent in COVID-19 (4–37%). This study evaluated the incidence and risk factors of acute kidney injury (AKI) in hospitalized patients with COVID-19.

**Methodology:** This study represents a prospective cohort in a public and tertiary university hospital in São Paulo, Brazil, during the first 90 days of the COVID-19 pandemic, with patients followed up until the clinical outcome (discharge or death).

**Results:** There were 101 patients hospitalized with COVID-19, of which 51.9% were admitted to the intensive care unit (ICU). The overall AKI incidence was 50%; 36.8% had hematuria or proteinuria (66.6% of those with AKI), 10.2% had rhabdomyolysis, and mortality was 36.6%. Of the ICU patients, AKI occurred in 77.3% and the mortality was 65.4%. The mean time for the AKI diagnosis was 6 ± 2 days, and Kidney Disease Improving Global Outcomes (KDIGO) stage 3 AKI was the most frequent (58.9%). Acute renal replacement therapy was indicated in 61.5% of patients. The factors associated with AKI were obesity [odds ratio (OR) 1.98, 95% confidence interval (CI) 1.04–2.76, *p* < 0.05] and the APACHE II score (OR 1.97, 95% CI 1.08–2.64, *p* < 0.05). Mortality was higher in the elderly (OR 1.03, 95% CI 1.01–1.66, *p* < 0.05), in those with the highest APACHE II score (OR 1.08, 95% CI 1.02–1.98, *p* < 0.05), and in the presence of KDIGO stage 3 AKI (OR 1.11, 95% CI 1.05–2.57, *p* < 0.05).

**Conclusion:** AKI associated with severe COVID-19 in this Brazilian cohort was more frequent than Chinese, European, and North American data, and the risk factors associated with its development were obesity and higher APACHE II scores. Mortality was high, mainly in elderly patients, in those with a more severe disease manifestation, and in those who developed KDIGO stage 3 AKI.

## Highlights

- Renal involvement in COVID-19 is frequent, especially in severe forms of this disease.
- In critically ill patients, obesity and higher APACHE II scores were risk factors for the development of AKI.
- ICU mortality was higher in the elderly, in patients with higher APACHE II scores, and in those with severe AKI.
- Of the critically ill patients with AKI, 61.5% had an indication for acute renal replacement therapy, and early indication (based on the inflammatory storm or cumulative water balance >3% of the weight) was associated with better survival.

## Introduction

In December 2019, an outbreak of unexplained pneumonia cases was reported in the city of Wuhan, China. Subsequently, the pathogen was identified as a new strain of coronavirus called severe acute respiratory syndrome coronavirus 2 (SARS-CoV-2) (1, 2). The World Health Organization (WHO) called the disease caused by this virus COVID-19, and in March 2020, it was declared a pandemic. The first case in Brazil was confirmed in February 2020; since then, the number of cases in the country has markedly increased. The clinical spectrum of COVID-19 is broad and, although diffuse alveolar damage and acute respiratory failure are the main characteristics of the disease in its severe form, renal involvement is frequent, especially among critically ill patients (20–40%), and is a factor related to worse outcomes (3, 4). Among the pathophysiological mechanisms involved, the virus has a cytopathic effect on the tubular epithelium and podocytes, in addition to a systemic inflammatory response associated with acute respiratory distress syndrome (crosstalk), rhabdomyolysis, and cytokine storm (5). Latin American data are scarce; hence, the objective of this study was to evaluate the incidence of acute kidney injury (AKI) in hospitalized patients diagnosed with COVID-19 and to identify both the risk factors associated with its onset and those associated with its prognosis during the first 90 days of the pandemic in a Brazilian public and tertiary university hospital.

## Methodology

A prospective cohort study of hospitalized patients diagnosed with COVID-19, confirmed by real-time polymerase chain reaction (RT-PCR) for SARS-Cov-2, was performed in clinical wards and intensive care units (ICUs) of a public and tertiary university hospital in São Paulo, Brazil, beginning 25 March 2020. Patients were followed from their hospitalization until the clinical outcome (discharge or death), the AKI diagnosis was assessed, and their risk factors were identified through the collection of information in electronic medical records. Renal function was evaluated daily by measuring serum creatinine and checking urine output, with an AKI diagnosis by the Kidney Disease Improving Global Outcomes (KDIGO) criteria (6): an increase of >0.3 mg/dl in baseline creatinine over 48 h; a 1.5-fold increase in baseline creatinine within 7 days; or a reduction in urinary output to <0.5 ml/kg/h in 6 h. Patients were classified into stage 1, 2, or 3 according to the increase in creatinine from baseline or reduction in urine output (6). Patients with chronic kidney disease stages IV and V, kidney transplant patients, and individuals under 18 years old were excluded.

This study was registered in the Brazilian Registry of clinical trials (ReBEC) under number RBR-62y3h7 and was approved by the Research Ethics Committee of Botucatu School of Medicine (CAAE 30451520.6.0000.5411). All the research was performed following current regulations, and written informed consent was obtained from all participants or their legal guardians.

The data were entered into an electronic spreadsheet, and any typing errors were eliminated. The analysis was performed with the aid of IBM SPSS 20 or Sigma Stat 3.5. Frequency or central tendency and dispersion measures were calculated for the categorical or continuous variables, respectively, and AKI, death, and acute renal replacement therapy indication were established as outcome variables.

The chi-squared test was used to compare categorical variables, while Student's *t*-test was used to compare continuous variables. Subsequently, through the construction of a logistic regression model, multivariate analysis was performed with odds ratio (OR) calculations, including all independent variables that showed association with the outcome, with *p* ≤ 0.20.

## Results

During the first 90 days of the pandemic, 101 patients with COVID-19 were hospitalized; 51.9% were admitted to the ICU and 48.1% were admitted to the wards. The overall AKI incidence was 50%; it was more frequent among patients admitted to the ICU than those in wards (77.3 vs. 20.4%, *p* < 0.01). The mean time for the AKI diagnosis was 7.2 ± 2 days, and KDIGO stage 3 AKI was the most frequent (49.1%), followed by stage 1 (27%). Upon hospital admission, 36.8% of patients had hematuria or proteinuria and 10.2% had rhabdomyolysis. Acute renal replacement therapy was indicated in 25 patients (49% of patients with AKI).

Hematuria or proteinuria at admission occurred in 66.6% of patients with AKI. The factors associated with the development of AKI (Table 1) were mechanical ventilation (17.6 vs. 76%, *p* < 0.0001), obesity (9.8 vs. 34%, *p* = 0.007), and ICU admission (23.5 vs. 80%, *p* < 0.0001).

**Table 1:** Clinical and laboratory characteristics of hospitalized patients regarding the presence or absence of AKI.

**Variables**	**General (*n* = 101)**	**Without AKI (*n* = 50)**	**With AKI (*n* = 51)**	***p*-value**
Male sex (%)	55 (54.4)	27 (52.9)	28 (56)	0.81
Age (years)^*^	57.89 ± 15.8	56.96 ± 16.9	61.62 ± 14.52	0.141
ACE inhibitors use (%)	45 (44.5)	20 (39.2)	25 (50)	0.373
Arterial hypertension (%)	54 (53.4)	25 (49)	29 (58)	0.48
Diabetes mellitus (%)	34 (33.6)	16 (31.4)	18 (7)	0.77
Obesity (%)	**22 (21.7)**	**5 (9.8)**	**17** **(****8****)**	**0.007**
CKD (%)	10 (9.9)	6 (11.8)	4 (9)	0.76
CVD (%)	19 (18.8)	8 (15.7)	11 (10)	0.57
Diuretic use (%)	29 (28.7)	12 (23.5)	17 (8)	0.346
GFR (ml/min/1.73 m^2^)^*^	86.54 ± 22.9	90.53 ± 26.97	81.55 ± 20.53	0.125
Mechanical ventilation (%)	**47 (46.5)**	**9 (17.6)**	**38 (76)**	** <0.0001**
Vasoactive drugs (%)	**47 (46.5)**	**9 (17.6)**	**38 (76)**	** <0.0001**
ICU admission (%)	**52 (51.4)**	**12 (23.5)**	**40 (80)**	** <0.0001**
Death (%)	**37 (36.6)**	**3 (5.9)**	**34 (68)**	** <0.0001**

*AKI, acute kidney injury; ACE, angiotensin-converting enzyme; CKD, chronic kidney disease; CVD, cardiovascular disease; GFR, glomerular filtration rate (CKD-EPI); ICU, intensive care unit*.

* *Mean ± SD. Bold values refer to variables that had a p-value less than 0.05*.

The overall mortality was 36.6% (37 patients); it was higher in ICU patients (28.1 vs. 91.9%, *p* < 0.0001). The factors associated with mortality outcome (Table 2) were the independent variables presence of cardiovascular disease (10.9 vs. 32.4%, *p* = 0.016), obesity (14.1 vs. 35.1%, *p* = 0.026), the need for mechanical ventilation (23.4 vs. 86.5%, *p* < 0.0001), and the presence of AKI (25 vs. 91.9%, *p* < 0.0001). Obesity was defined by WHO by body mass index (BMI) ≥30 kg/m^2^.

**Table 2:** Clinical and laboratory characteristics of hospitalized patients according to outcome.

**Variables**	**No death (*n* = 64)**	**Death (*n* = 37)**	***p*-value**
Male sex (%)	35 (54.68)	20 (54.05)	0.53
Age (years)^*^	**57.07** **±** **16.57**	**63.05** **±** **13.97**	**0.048**
Arterial hypertension (%)	30 (46.9)	24 (64.9)	0.124
Diabetes mellitus (%)	17 (26.6)	17 (45.9)	0.077
CKD (%)	5 (7.8)	5 (13.5)	0.56
CVD (%)	**7 (10.9)**	**12 (32.4)**	**0.016**
Diuretic use (%)	15 (23.4)	14 (37.8)	0.189
GFR (ml/min/1.73 m^2^) on admission^*^	**96.2** **±** **22.25**	**79.72** **±** **23.15**	**0.006**
Mechanical ventilation (%)	**15 (23.4)**	**32 (86.5)**	** <0.0001**
Vasoactive drugs (%)	**15 (23.4)**	**32 (86.5)**	** <0.0001**
ICU admission (%)	**18 (28.1)**	**34 (91.9)**	** <0.0001**
AKI (%)	**16 (25)**	**34 (91.9)**	<0.0001
APACHE II^*^	**16.11** **±** **4.39**	**23.61** **±** **5.67**	** <0.001**
SOFA^**^	**7.5 (2–9)**	**11 (9–13)**	** <0.001**

*AKI, acute kidney injury; CKD, chronic kidney disease; CVD, cardiovascular disease; GFR, glomerular filtration rate (CKD-EPI); ICU, intensive care unit; APACHE II, Acute Physiology and Chronic Health Evaluation score; SOFA, Sequential Organ Failure Assessment score*.

* *Mean ± SD*;

** *median (IQR). Bold values refer to variables that had a p-value less than 0.05*.

Based on logistic regression analysis, mechanical ventilation [OR 2.23, 95% confidence interval (CI) 1.09–2.97, *p* < 0.01] and age (OR 1.05, 95% CI 1.01–1.08, *p* = 0.04) were AKI risk factors. Cardiovascular disease (OR 1.01, 95% CI 1.001–1.273, *p* = 0.006), the presence of AKI (OR 1.04, 95% CI 1.003–1.464, *p* = 0.01), and mechanical ventilation (OR 1.015, 95% CI 1.001–1.33, *p* = 0.008) were variables associated with death (Table 3).

**Table 3:** Logistic regression of variables associated with AKI and death in hospitalized patients.

**Variables**	**OR**	**95% CI**	***p*-value**
**AKI**			
Age	**1.05**	**1.01–1.08**	**0.04**
Obesity	1.05	0.99–1.19	0.07
Mechanical ventilation	**2.23**	**1.09–2.97**	** <0.01**
Arterial hypertension	1.03	0.99–1.23	0.08
**Death**			
Obesity	1.28	1.04–11.52	0.082
Mechanical ventilation	**1.015**	**1.001–1.33**	**0.008**
AKI	**1.04**	**1.003–1.464**	**0.01**
CVD	**1.01**	**1.001–1.273**	**0.006**
Diabetes mellitus	1.142	0.99–1.59	0.11
Age	1.033	0.95–1.11	0.39
GFR	1.012	0.97–1.054	0.55

*CI, confidence interval; AKI, acute kidney injury; CVD, cardiovascular disease; GFR, glomerular filtration rate (CKD-EPI). Bold values refer to variables that had a p-value less than 0.05*.

When analyzing only patients admitted to the ICU, AKI occurred in 77.3% of patients, and overall mortality was 65.4%. The mean time for the AKI diagnosis was 6 ± 2 days, and KDIGO stage 3 AKI was the most frequent (58.9%). Acute renal replacement therapy was indicated in 61.5% of the patients.

The factors associated with AKI development in ICU patients (Table 4) were obesity (7.6 vs. 38.5%, *p* = 0.049), the use of corticosteroids (38 vs. 17.9%, *p* = 0.04), and APACHE II (15.3 ± 4.8 vs. 22.7 ± 5.7, *p* < 0.001) and SOFA (6.3 ± 2.9 vs. 10.2 ± 3.1, *p* = 0.003) scores.

**Table 4:** Clinical and laboratory characteristics of patients admitted in ICU regarding the presence or absence of AKI.

**Variables**	**General (*n* = 52)**	**Without AKI (*n* = 12)**	**With AKI (*n* = 40)**	***p*-value**
Male sex (%)	24 (46.1)	4 (36.5)	20 (52.0)	0.31
Mechanical ventilation (%)	49 (94.2)	11 (84.6)	38 (97.4)	0.59
ACE inhibitors use (%)	17 (32.7)	3 (29.8)	14 (46.9)	0.16
Arterial hypertension (%)	23 (44.2)	5 (45.4)	18 (57.7)	0.48
Diabetes mellitus (%)	25 (48)	6 (54)	19 (48.1)	0.61
Obesity (%)	**16 (30.7)**	**1 (7.6)**	**15 (38.5)**	**0.049**
Age (years)^*^	59.82 ± 14.4	58.8 ± 17.4	59.8 ± 13.5	0.14
Basal creatinine (mg/dl)^*^	0.84 ± 0.34	0.82 ± 0.33	0.88 ± 0.35	0.65
Corticosteroids use (%)	**12 (23)**	**5 (38)**	**7 (17.9)**	**0.04**
Chloroquine use (%)	5 (9.6)	2 (15.4)	3 (7.6)	0.76
APACHE II^*^	**19.8** **±** **5.3**	**15.3** **±** **4.8**	**22.7** **±** **5.7**	** <0.001**
SOFA^*^	**8.6** **±** **3.0**	**6.3** **±** **2.9**	**10.2** **±** **3.1**	**0.003**

*AKI, acute kidney injury; ACE, angiotensin-converting enzyme; ICU, intensive care unit; APACHE II, Acute Physiology and Chronic Health disease Classification System II score; SOFA, Sequential Organ Failure Assessment score*.

* *Mean ± SD. Bold values refer to variables that had a p-value less than 0.05*.

The mortality of ICU patients was 65.4%; it was higher in older patients (53.2 ± 9.3 vs. 61.8 ± 11.9, *p* = 0.04), in those with AKI (33.3 vs. 97%, *p* = 0.04), in those who received acute renal replacement therapy (21.4 vs. 55.3%, *p* = 0.04), and with higher APACHE II (11.1 ± 4.7 vs. 23.3 ± 11.8, *p* < 0.001) and SOFA (6.1 ± 3.1 vs. 10.3 ± 2.9, *p* = 0.003) scores (Table 5).

**Table 5:** Clinical and laboratory characteristics in patients admitted in ICU according to outcome.

**Variables**	**No death (*n* = 18)**	**Death (*n* = 34)**	***p*-value**
Male sex (%)	5 (11)	20 (52.6)	0.24
Comorbidity (%)	9 (64)	33 (86.9)	0.12
Age (years)^*^	**53.2** **±** **9.3**	**61.8** **±** **11.9**	**0.04**
AKI (%)	**6 (33.3)**	**33 (97)**	**0.01**
KDIGO 3 (%)	**0 (0)**	**22 (57.9)**	** <0.001**
Renal acute support (%)	**3 (21.4)**	**21 (55.3)**	**0.04**
APACHE II^*^	**11.1** **±** **4.7**	**23.3** **±** **11.8**	** <0.001**
SOFA^*^	**6.1** **±** **3.1**	**10.3** **±** **2.9**	**0.003**

*AKI, acute kidney injury; ICU, intensive care unit; APACHE II, Acute Physiology and Chronic Health Evaluation score; SOFA, Sequential Organ Failure Assessment score*.

* *Mean ± SD. Bold values refer to variables that had a p-value less than 0.05*.

In the logistic regression analysis, the AKI risk factors were obesity (OR 1.98, 95% CI 1.04–2.76, *p* < 0.05) and the APACHE II score (OR 1.97, 95% CI 1.08–2.64, *p* < 0.05). The variables associated with death were age (OR 1.03, 95% CI 1.01–1.66, *p* < 0.05), a higher APACHE II score (OR 1.08, 95% CI 1.02–1.98, *p* < 0.05), and KDIGO stage 3 AKI (OR 1.11, 95% CI 1.05–2.57, *p* < 0.05; Table 6).

**Table 6:** Logistic regression of variables associated with AKI and death in patients admitted in ICU.

**Variables**	**OR**	**95% CI**	***p*-value**
**AKI**			
Obesity	**1.98**	**1.04–2.76**	** <0.05**
Corticosteroids use	0.97	0.96–1.23	0.13
APACHE II	**1.97**	**1.08–2.64**	** <0.05**
**Death**			
Age	**1.03**	**1.01–1.66**	** <0.05**
APACHE II	**1.08**	**1.02–1.98**	** <0.05**
KDIGO 3	**1.11**	**1.05–2.57**	** <0.05**

*CI, confidence interval; AKI, acute kidney injury; ICU, intensive care unit; APACHE II, Acute Physiology and Chronic Health disease Classification System II score. Bold values refer to variables that had a p-value less than 0.05*.

When we evaluated only patients submitted to acute renal replacement therapy, the standard timing for initiation (gap demand vs. capacity or urgency for dialysis) occurred in 15 patients (60%), and early indication based on the presence of AKI stage 1 or 2 and cytokine storm (continuous fever higher than 39°C) or cumulative positive water balance higher than 3% of weight occurred in 10 patients (40%). There was no difference between the standard and early groups regarding mechanical ventilation, vasoactive drug use, age, and prognostic scores (APACHE II and SOFA), but there was a difference in mortality (100 vs. 70%, *p* < 0.05; Table 7).

**Table 7:** Clinical and laboratory characteristics in patients admitted in ICU according to renal acute support indication.

**Variables**	**General (*n* = 25)**	**Standard indication (*n* = 15)**	**Early indication (*n* = 10)**	***p*-value**
Male sex (%)	12 (48)	7 (45.4)	5 (50)	0.45
Age (years)^*^	59.2 ± 13.8	60.2 ± 13.3	57.8 ± 13.9	0.62
Arterial hypertension (%)	10 (40)	5 (36.4)	5 (50)	0.45
Diabetes mellitus (%)	10 (40)	7 (45.4)	3 (30.0)	0.06
Obesity (%)	14 (56)	8 (54.5)	6 (60)	0.81
ATN-ISS^*^	0.65 ± 0.13	0.68 ± 0.13	0.61 ± 0.14	0.36
APACHE II^*^	22.1 ± 8.1	23.5 ± 8.2	20.2 ± 8.1	0.09
**Death (%)**	**22 (88)**	**15 (100)**	**7 (70)**	**0.05**

*AKI, acute kidney injury; ICU, intensive care unit; ATN-ISS, Acute Tubular Necrosis–Individual Severity Score; APACHE II, Acute Physiology and Chronic Health disease Classification System II score*.

* *Mean ± SD. Bold values refer to variables that had a p-value less than 0.05*.

## Discussion

This study describes the first 90 days of the COVID-19 pandemic in a public university hospital in the interior of São Paulo, Brazil, which is a reference for 28 municipalities in the region with more than 2 million inhabitants. During this period, 101 patients with a COVID-19 diagnosis were hospitalized, with 51.9% admitted to the ICU and 48.1% admitted to the ward. The overall AKI incidence was 50%, with an average diagnosis time of 7 days. This AKI incidence is higher than that reported to date in the literature. Chinese studies (9, 12–19) have reported a low and variable AKI incidence (0.5–7%), higher in severe COVID-19 cases (2.9–19%). European and North American studies (20–22) have reported an AKI incidence of 20–40% of patients hospitalized with COVID-19. In all cohorts, AKI occurred between the 7th and the 14th day of illness; it was associated with higher hospital mortality and decisive in the prognosis of these patients (9, 12–23). Brazil is a large country with several vulnerable groups, in addition to an emerging economy and fragile social protection, which may have contributed to more demand for health services and increased development of serious forms of COVID-19 (10).

The higher AKI incidence in western countries could be associated with the higher expression of angiotensin-converting enzyme 2 (ACE2) in podocytes and proximal tubule in western compared with eastern individuals, as identified in normal kidneys and described by Pan et al. (24) However, other studies did not find the SARS-CoV-2 virus in the renal biopsy/autopsy issue samples (25, 26). SARS-CoV-2 has a protein, called Spike (S), which binds to the ACE2 receptor present in host cells, enabling its activation and cleavage by transmembrane proteases and culminating in the release of fusion peptides by the virus. ACE2 is highly expressed in the mouth and tongue, in addition to alveolar epithelial cells. In the kidneys, it is highly expressed in proximal tubule cells and to a lesser extent in podocytes (15, 27).

AKI is a complex disorder characterized by an abrupt reduction in renal function, usually followed by accumulation of nitrogen products, electrolyte imbalance, and volume overload (28). Its incidence in hospitalized patients varies between 5 and 7%; it is higher in ICU patients, around 50%. Despite the technological advances that have occurred and the reduction in the mortality rate in the last decade, the AKI prognosis remains severe and the death rate remains high, especially in patients in need of dialysis (up to 62%) (29–33).

In logistic regression, we identified that the factors associated with the development of AKI in patients hospitalized with COVID-19 were mechanical ventilation and older age. In the case of patients with COVID-19 admitted to the ICU, the factors associated with the development of AKI were obesity and higher APACHE II scores. Obesity represents a risk factor for greater severity and worse prognosis in COVID-19. This is due to the intensification of the individual's inflammatory state (dysregulation of adipokines and greater release of tumor necrosis factor alpha and interleukin 6), compromised immune response, increased thrombotic risk, and negative impacts on pulmonary mechanics (decreased forced expiratory reserve volume, functional capacity, and respiratory compliance). In addition, there are ACE2 receptors in adipose tissue, and thus that tissue is an important viral reservoir (34, 35).

In a Brazilian study, Bucuvic et al. (36) reported that 62% of patients diagnosed with AKI were male, 65.2% were over 60 years old, 61.9% had diabetes mellitus, 44.4% were hypertensive, and 21.9% had CKD. Garcia et al. (37) described that 62% of those admitted to the ICU with AKI were male, 51.5% were over 60 years old, 57.7% of patients had arterial hypertension, 27.4% were cardiac patients, and 26.6% had diabetes mellitus. Santos and Matos (38) compared patients who did and did not develop AKI in the ICU. Patients affected by AKI were older (56.4 ± 18.8 vs. 46.8 ± 16.5 years, *p* = 0.0028), and more presented septic shock (19.2 vs. 6.5%, *p* < 0.05) or sepsis (17.3 vs. 3.9%, *p* = 0.012).

We already know from the non-COVID-19 literature that AKI is associated with worse clinical outcomes. An international multicenter study from 2015 with 1032 ICU patients showed that AKI was independently associated with higher mortality at all stages, with the following odds ratios: 1.7 for KDIGO stage 1 and 6.9 for KDIGO stage 3. In ICU patients, AKI is also associated with longer duration of mechanical ventilation, use of vasoactive drugs, and increased length of hospital stay, with acute renal replacement therapy being necessary in 50% of cases.

The data presented by our study identified an overall mortality of 37.2%; it was higher in ICU patients (28.1 vs. 91.9%, *p* < 0.0001). The factors associated with mortality were the presence of comorbidities such as cardiovascular disease, the presence of AKI, and mechanical ventilation. Mortality was 65.4% in ICU patients, higher in individuals with older age, higher APACHE II score, and KDIGO stage 3 AKI.

Upon hospital admission, 36.8% of patients had hematuria and/or proteinuria, and 66.6% of patients were diagnosed with AKI. Data published to date (9, 12–23) have reported that at COVID-19 diagnosis, 27–64% of the patients presented hematuria and/or proteinuria. However, it is not known whether they were due to AKI or previous chronic kidney disease, because creatinine values at admission were considered as the baseline, and creatinine values were not assessed before or after hospital admission (19).

Acute renal replacement therapy was indicated in 61.5% of patients with AKI in the ICU setting. When we evaluated only patients submitted to acute renal replacement therapy, the standard indication (demand gap vs. capacity) occurred in 60% of patients and the early indication, based on the presence of cytokine storm (continuous fever higher than 39°C) or positive cumulative water balance higher than 3% of weight, occurred in 40% of patients. It is known that the time to indicate acute renal replacement therapy to a patient with AKI is based on the benefits and risks that therapy can bring. Dialysis can control fluid, electrolyte, and acid–base status and remove uremic toxins. On the other hand, it also removes medications and can cause bleeding or infections, and in the case of hemodialysis, it can increase the risk of hemodynamic instability and oxidative stress induced by dialyzers (7, 8, 11). Studies, including clinical trials and meta-analyses, have shown no benefit in the early indication of dialysis based on laboratory criteria, length of stay or ICU admission, or AKI stage. They have concluded that the indication for acute renal replacement therapy should be performed when criteria specify the urgency for dialysis—such as metabolic acidosis, refractory electrolyte disorders or hypervolemia, exogenous intoxications by drugs that may be removed by dialysis, and/or uremic syndrome—or, earlier, when there is a gap between the patient's demand and his or her renal capacity (39–43). However, early indication could be beneficial, especially in patients with severe acidosis, acute respiratory distress syndrome, and fluid overload (39).

In our study, there was no difference between the standard and early groups regarding mechanical ventilation, vasoactive drug use, age, and prognostic indexes (APACHE II and SOFA), but there was a difference in mortality (100 vs. 70%, *p* < 0.05). The lower mortality in earlier indication for acute renal replacement therapy (RRT) has a pathophysiological rationale, as described by Ronco and Reis (5) that the removal of cytokines at the time of the inflammatory storm could reduce the magnitude of a renal lesion, as well as pulmonary complications and hypercoagulability, but the role of cytokine removal by RRT is still unclear at this time. All patients who underwent acute RRT in our study did so by continuous venous therapy (hemodiafiltration or continuous hemofiltration) or prolonged hemodialysis with high permeability capillary.

This study has some limitations: the sample size is small, the data were collected from a single center, and cytokine removal during acute RRT was not calculated. However, this study represents initial data on the epidemiological profile of AKI associated with COVID-19 in Brazil, a country with continental characteristics and a heterogeneous and vulnerable population. It should serve as an impetus for new and promising research endeavors.

## Conclusion

AKI associated with severe COVID-19 in Brazilian patients was much more frequent than data already published from Chinese, European, and North American cohorts, with obesity and higher APACHE II scores being the risk factors associated with AKI development. Mortality was high in this population, especially elderly patients, and in those with greater clinical severity and who develop KDIGO stage 3 AKI. When indicated early, acute renal replacement therapy may be associated with better survival by removing cytokines or excess fluids. More and larger studies are needed to understand the role of early indication for acute renal replacement therapy in the prognosis of patients with severe COVID-19.

## Data Availability Statement

The raw data supporting the conclusions of this article will be made available by the authors, without undue reservation.

## Ethics Statement

The studies involving human participants were reviewed and approved by Research Ethics Committee of Botucatu School of Medicine (CAAE 30451520.6.0000.5411). The patients/participants provided their written informed consent to participate in this study.

## Author Contributions

All authors made substantial contributions to conception and design, acquisition of data, and/or analysis and interpretation of data, took part in drafting the article or revising it critically for important intellectual content, gave final approval of the version to be published, and agree to be accountable for all aspects of the work.

## Conflict of Interest

The authors declare that the research was conducted in the absence of any commercial or financial relationships that could be construed as a potential conflict of interest.
